# Presence, Absence, and Alterity: *Fire Space* and Goffman’s *Selves* in Postdigital Education

**DOI:** 10.1007/s42438-021-00265-1

**Published:** 2021-10-30

**Authors:** Lesley Gourlay

**Affiliations:** University College London Institute of Education, London, UK

**Keywords:** Digital higher education, Video conferencing, Learning spaces, Learnification, Social topologies, Fire space, Goffman's lecturer selves

## Abstract

The literature on space in higher education has arguably been dominated by the concept of ‘learning spaces’. In this paper, I will argue that this construct, while appearing student-focused and creative, is ideologically circumscribed by an underlying social constructivism. Following Bayne et al. (2014), I draw on science and technology studies to consider social topologies, in particular regional space, *network space*, and their proposed *fluid space*, and the work of Law and colleagues on the category of *fire space*, derived from Bachelard’s (*The Psychoanalysis of Fire*, 1964) disquisition on the nature of fire. I work with this construct in an analysis of postdigital education, in particular looking at synchronous interaction via video conferencing software such as Zoom. Linking this analysis to the work of Goffman and his concept of the lecturer *selves* (Goffman in *Forms of Talk*, 1981), I argue that the concept of *fire space* may allow for a more nuanced and accurate account of the flickering, contingent nature of (co) presence, absence, and alterity, allowing for a more immanent account of digital interaction in ‘distance’ or ‘online’ education.

## Introduction

The literature on space in relation to postdigital higher education, and indeed higher education more broadly, has arguably been dominated by the concept of ‘learning spaces’. In this paper, I will argue that this construct, while appearing student-focused and creative, is ideologically circumscribed by an underlying social constructivism, a fundamentally performative framing, and an ethos of ‘learnification’ (Biesta 2012), despite the welcome influence of sociomaterial perspectives in recent years (e.g. Fenwick et al. 2011) and related recognition of nonhuman actors. In this critique, I will pay particular attention to the effects of using ‘learning’ as an adjective, and I will propose that there is a danger the generative insights of posthumanism and sociomateriality may be lost if subsumed under a pragmatic, ‘what works’ discourse, or if they are merely added to existing notions of ‘learning space’. Instead I argue that more fundamental shifts in theoretical focus should ensue. Following Bayne et al. (2014), I draw on Mol and Law (1994) to consider social topologies, in particular regional space, *network space*, and their proposed *fluid space*. I then review Law and Mol’s (2001) discussion of the additional category of ‘fire space’, which they derive from Bachelard’s (1964) disquisition on the nature of fire. In a challenging and seemingly tangential move, Law and Mol draw three points from Bachelard. The first point is that, for them, the trope of death and rebirth can be seen ‘…as a metaphor for treating *the continuity of shape as an effect of discontinuity*…, and that in a topology of fire, constancy is produced in abrupt and discontinuous movements’ (Law and Mol 2001: 615). Their second point is that Bachelard’s analysis can be seen as ‘…a call for attending to discontinuous transformation as a flickering relation between presence and absence’; they urge us to see fire as ‘…a metaphor for thinking about the dependence of that which cannot be made present on that which is absent’. They go on to propose that ‘… in fire space a shape achieves constancy in a relation between presence and absence: the constancy of object presence depends on simultaneous absence or alterity’. Their third point refers to Bachelard’s discussion of the ‘star pattern’ of reverie, which, they argue ‘…evokes a specific version of the relation between presence and absence: a link between a single present centre and multiple absent Others’ (*loc cit*). The notion of fire object is also Their third point refers to Bachelard’so proposed in Law and Singleton (2005). In this paper, I will attempt to work with this construct in an analysis of postdigital education, in particular looking at synchronous interactions via video conferencing software such as Zoom. In my discussion, I also link this analysis to the work of Goffman and his concept of the lecturer *selves* (Goffman 1981). I will argue that the concept of fire space may allow for a more nuanced and accurate account of the flickering, contingent nature of (co) presence, absence and alterity, allowing for a more immanent account of digital interaction in ‘distance’ or ‘online’ education, in what Law and Mol characterise as ‘…patterns of conjoined alterity’ (*loc cit*). This will be linked to the notion of selves in terms of not only verbal and textual performance as Goffman sets out, but also in terms of presence, absence, and alterity of the embodied self, image, representation and voice, in an attempt to extend Goffman’s analysis to address the nature of self and presence in postdigital settings. I will conclude by arguing that theorisations of space which turn the focus away from ‘learning’ in postdigital education may offer the field a more granular view of the flickering, ephemeral, and at times uncanny nature of these encounters.

## The Problem with ‘Learning Spaces’

The term ‘learning spaces’ has become commonplace in higher education over recent years, with reference to the ways in which aspects of the physical campus might influence student engagement. In an early review, Temple and Filippakou (2007) discusses the issue, including guidance surrounding the use of space in universities. JISC (2006) provided the following definition:

A learning space should be able to motivate learners and promote learning as an activity, support collaboration as well as formal practice, provide a personalised and inclusive environment, and be flexible in the face of changing needs. The part technology plays in achieving these aims is the focus of this guide (JISC 2006: 2).

The use of the term ‘learning space’ is used here and is prevalent in the sector. In Gourlay and Oliver (2018), we discuss this document in detail, pointing out that one of the features of the term ‘learning spaces’ is that it is not generally used to refer to classrooms, but more to public areas outside the classroom, such as a ‘learning cafe’ or another space designated for student collaborative working. Learning spaces are explicitly defined as a replacement for the previously dominant ‘teaching spaces’, which are described as retrograde. In the 2018 critique, we made the point that the way the concept is portrayed by JISC serves to de-emphasise teaching, instead promoting small group interaction outside of formal instruction as the desired mode of engagement for students, with a strong emphasis on observable interaction. We also pointed out the way in which ‘learning spaces’ are enrolled in an effort to make the university appealing, with the students positioned as customers whose attention must be competed for via a range of material commodities and a ‘proactive service-delivery culture’. Digital technologies are ubiquitous in this imaginary, but how they are used in terms of scholarship (rather than information retrieval) is not explored. We also analysed a more recent guide to ‘learning space’ (UCISA 2016) that refers to the concept of ‘built pedagogy’ (Monaghan 2002), defined as ‘…architectural embodiments of educational philosophies’, based on the assumption that ‘… the way in which a space is designed shapes the learning that takes place in that space’ (UCISA 2016: 9). In both of these guides, a strongly deterministic relationship is assumed between the form of the space and the resultant ‘learning’, for which observable interaction seems to stand as a proxy.

In terms of digital technology in education, this stands in contrast with what Law and Singleton (2005) called the ‘incorporeal fallacy’, the idea that materiality and bodies somehow disappear in the realm of the digital, in a fantasy of untrammelled freedom from the constraints they represent. This assumption surrounding the nature of space has, however, been challenged in the literature, as the complex, co-constitutive, and sociomaterial nature of education practices has become more recognised (e.g. Gourlay and Oliver 2018; Acton 2017), alongside the growing influence of the *mobilities* paradigm (Sheller and Urry 2006; Hannam et al. 2006; Urry 2007), on research into higher education (e.g. Enriquez 2011, 2013; Edwards et al. 2011; Hamilton and Friesen 2013; Bayne et al. 2014), plus work more broadly on the nature of digital objects (e.g. Adams and Thompson 2016). These bodies of work provide growing insights into the sheer complexity, contingency, and shifting nature of educational practices and engagement in terms of spatiality, embodiment, and movement. These are crucial questions for scholars of higher education when considering both the campus, and also the nature of online engagement.

In foundational work focused on what was at the time called ‘computer conferencing’, the related question of ‘presence’ has been explored, in particular how presence can be understood when individuals are not co-present in a physical, face-to-face setting. Garrison and colleagues explored the various dimensions of presence (Garrison et al. 2000, 2001), with a later paper pointing out that ‘interaction is not enough’ to create a sense of presence (Garrison and Cleveland-Innes 2005). However, given the centrality of the intersection of space and presence to remote digital education, arguably, it has not been adequately theorised in the literature. An exception is Bayne et al. (2014), who draw on theoretical work in science and technology studies focused on the concept of *topology*, in an insightful discussion of the nature of ‘distance’ learning and various conceptions of the campus held by remote students. I seek to build on this work by drawing on the same set of theoretical resources, in order to relate this to questions around the complex and multifaceted nature of space and presence in digital education.

## Social Topologies

Mol and Law (1994), in a consideration of the medical condition anaemia, discuss ‘social topology’, which they define as follows:

Unlike anatomy, topology doesn’t localise objects in terms of a given set of co-ordinates. Instead, it articulates *different rules for localising* in a variety of coordinate systems. This it doesn’t limit to the three standard axes, X, Y and Z, but invents alternative systems of axes. In each of these, another set of mathematical operations is permitted which generates its own ‘points’ and ‘lines’. These do not necessarily map on to those generated in an alternative axial system. Even the activity of ‘mapping’ itself differs between one space and another. Topology, in short, extends the possibilities of mathematics far beyond its original *Euclidean* restrictions by articulating other spaces. (Mol and Law 1994: 643)

They take this concept from mathematics and adapt it to social theory, based on the contention that ‘the social’ does not exist as a single spatial type. Rather, it performs several *kinds of space* in which different ‘operations’ take place. First, there are regions in which objects are clustered together and boundaries are drawn around each cluster. Second, there are networks in which distance is a function of the relations between the elements and difference a matter of relational variety (Mol and Law 1994: 643). However, they suggest that in addition to these two kinds of space, ‘[s]ometimes, we suggest, neither boundaries nor relations mark the difference between one place and another. Instead, sometimes boundaries come and go, allow leakage or disappear altogether, while relations transform themselves without fracture. Sometimes, then, social pace behaves like a *fluid*.’ (Mol and Law 1994: 643) Discussing the case of the prevalence of anaemia in the Netherlands in comparison with African nations, they point out the complications involved in the measurement of the condition. From an actor-network point of view, ‘…the space in which regions can be drawn and differentiated exists. But it doesn’t exist in the order of things. Rather, it is an effect or a product which depends on another quite different kind of space, the space of networks. This isn’t regional in character, but is generated within a *network topology*.’ (Mol and Law 1994: 649) Discussing the nature of network topology, they point out that proximity is not metric, and that ‘… “here” and “there” are not objects or attributes that lie inside or outside a set of boundaries… places with a similar et of elements and similar relationships between them are lose to one another, and those with different elements or relations are far apart’. (Mol and Law 1994: 649) They use the example of two haemoglobin meters which may be geographical distant, but are close in a network topology, an inter-topological effect when one topology meets another, in a ‘fold’ of regional surfaces (Cooper 1992).

However, they point out that this type of fold can only take place ‘if the network holds’, giving the example of diagnostic medical labs in the Netherlands in comparison with those in Africa. The measurement is not in fact immutable. ‘The folded surface of the region starts to flatten out, and the space–time tunnel of the network dissolves.’ (Mol and Law 1994: 652) They provide a detailed discussion of the ways in which anaemia is diagnosed in the Netherlands and also in clinics in southern Africa, focusing on the contrast between laboratory testing and clinical diagnosis using observation and questioning of the patient. They characterise these as two different networks, but crucially, they point out that they are not clearly separate. Instead, elements of both may be present in the different regions. As they put it, ‘[w]hat we are looking at is something different. We’re looking at *variation without boundaries and transformation without discontinuity*. We’re looking at flows. The space with which we’re dealing is *fluid*.’ (Mol and Law 1994: 658) They define fluid space as a topology which is neither regional nor network-based, there are other types of topology.

… there are others too, and one of them is fluid. For there are social objects which exist in, draw upon and recursively form fluid spaces that are defined by liquid continuity. Sometimes fluid spaces perform sharp boundaries. But sometimes they do not – though one object gives way to another. So there are mixtures and gradients. And inside these mixtures everything informs everything else – the world doesn’t collapse if some things suddenly fail to appear. (Mol and Law 1994: 659)

As they explain:

For in a network, things that go together depend on one another. If you take one away, the consequences are likely to be disastrous. But in a fluid, it isn’t like that as there is no ‘obligatory point of passage’; no place past which everything else has to file; no panopticon; no centre of translation; which means that *every* individual element may be superfluous. (Mol and Law 1994: 661)

They see these three types of topology as co-existing with each other in ‘intricate relations’.

In a related work which develops these ideas further, Law and Mol (2001) remind us of relationships between social studies of science and epistemology, in particular the role of early laboratory studies such as Latour and Woolgar (1979), Knorr-Cetina (1981), and Lynch (1985). As they point out, these studies of scientific practice were seen as a challenge to the notion of scientific universality, demonstrating that, ‘[q]uite quickly the argument was made: scientific findings and theories are made in specific locations. They are always made somewhere. In a locality. They are regional, not universal.’ (Law and Mol 2001: 610) In order for these to be regarded as ‘facts’, a configuration of facts and context must be held stable. The focus in the research on the work of holding configurations in shape with ‘immutable mobiles’ (Latour 1986) led to the actor-network theory. They give an example of a ship:

On the one hand it generates an immutable mobile, a vessel that made it safely across the seven seas, an *object* holding itself together in a particular web of relations. But it also, at the same time, implies a *form of spatiality*. The argument then, is that a network-object also implies a stable shape within a network space. The two go together. Spatiality is an aspect of network stability. A large network (with its wind, its stars, its merchants, and its princes) implies a network space which renders possible the immutable mobility of an object – such as a Portuguese ship travelling from Lisbon to Calicut. (Law and Mol 2001: 611–612)

In the case of the immutable mobile, there are two forms of spatiality: *Euclidean* and *network*.

In Cartesian, regional, or Euclidean space place is defined by a set of relative three-dimensional coordinates. So long as a ship is tied up in the harbour in Lisbon, it does not move. And as soon as it sets out to sea, it displaces itself. But the space implied in actor-network theory is different. Is there no change in the working relations between the hull, the spars, the sails, the sailors, and all the rest? If this is the case then the ship is immutable in the sense intended by Latour. It does not move in relation to a network space. (Law and Mol 2001: 612)

In *network space*, the vessel holds its shape, and holds its position in that space. It does not displace itself; instead, it is an *immutable immobile*. Relations are sustained in a stable manner. The mobility of the ships only exists in Euclidian space:

…the immutable mobile achieves its character by virtue of participation in two spaces: it participates in *both network and Euclidean space*…it is the *interference between the spatial systems that afford the vessel its special properties.* We are in the presence of two topological systems, two ways of performing space. And the two are being linked together. (*loc cit*)

Developing the notion of *fluid space*, they refer to De Laet’s and Mol’s (2000) study of bush pumps in Zimbabwe to give an example of something that does not move within a network but is other to the network and its spatialities, outside a network. It changes shape.

Of this pump and everything that allows it to work, nothing in particular necessarily holds in place. Bits break off the device and are replaced with bits which do not seem to fit. And other components - we are talking here both of parts of the ‘machine itself’, and of the social relations embedded in it – are added to it, components which were not in the original design itself. (Law and Mol 2001: 613)

In Euclidean and also network space, the bush pump is an object which changes shape. The pump shows configurational variance; it is a mutable mobile. It is a *fluid object* in a topological system *fluid spatiality*. For them, the defining feature of a fluid space is incremental change in shape, in which elements may be lost or added, with a continuity of function.

## Fire Space and Fire Objects

The philosopher Bachelard wrote about the nature of fire:

The fascinated individual hears the call of the funeral pyre. For him destruction is more than a change, it is a renewal. (Bachelard 1964: 13)

He writes about the reveries of a person who stares at a fire:

…the reverie is entirely different from the dream by the very fact that it is always more or less centred upon one object. The dream proceeds on its way in linear fashion, forgetting its original path as it hastens along. The reverie works in a star pattern. It returns to the centre to shoot out new beams. (Bachelard 1964:14)

Law and Mol (2001) take three points from this. The first is that the trope of death and rebirth ‘…as a metaphor for treating *the continuity of shape as an effect of discontinuity *… The difference is that, whereas in fluidity constancy depends on gradual change, in a topology of fire constancy is produced in abrupt and discontinuous movements.’ (Law and Mol 2001: 615) They characterise fire space as consisting of ‘…a flickering relation between absence and presence’, in which fire is used as a metaphor for the existence of elements which rely on the absence, or alterity, of others. A further attribute they identify relates to Bachelard’s notion of the ‘star pattern’ of reverie. They link this to a relation between absence and presence in which there exists ‘…a single present centre and multiple absent Others … a relatively stable set of star-like enactments between a single present and multiple absences’ (Law and Mol 2001).

Law and Singleton (2005) in a paper which explicitly builds on Law and Mol (2001) go on to develop this construct further in an analysis of treatment for alcoholic liver disease, which they propose as a *fire object*. In the course of their healthcare-related study, they found it difficult to keep the condition ‘in focus’, and suggested on that basis, that ‘social science methods are ill-adapted for the study of complex and messy objects’ (Law and Singleton 2005: 1). They refer to three types of object established in actor-network theory; *region*, *network*, and *fluid* in their discussion, and also the *fire* object, discussed above, which they define here as one which ‘treats objects as patterns of discontinuity between absence and presence’ (*loc cit*). They identify two strategies for ‘knowing mess’; epistemological and ontological. The former rests on the idea that messy objects are difficult to know because people have varied perspectives on them, are ‘interpretively complex’, and mean different things to different groups of people. This assumes there is a real object to be retrieved behind these interpretations. They point out that this is common in the study of objects in science and technology studies, such as in Star and Griesemer’s (1989) study of *boundary objects*. In contrast, Law and Singleton adopt an ontological perspective on objects, in which messy objects are ‘enacted into being’ (*loc cit*). Like Law and Mol, they discuss the concept of *immutable mobiles*, and also introduce de Laet and Mol’s (2000) concept of the *mutable mobile*, discussed above in terms of the bush water pump in rural Zimbabwe, which is itself constantly changing its form due to repairs and other aspects of its operation which change. They suggest it should be seen as a *fluid object*, which changes gradually and gently.

However, when they attempt to apply the concept of *fluid object* to understand the ontological nature of alcoholic liver disease, they find it resists that analysis. With reference to Mol’s (2002) book *The Body Multiple*, they highlight how the body is regarded as a single entity, but it is multiple ‘because it is enacted in multiple practices’ (Law and Singleton 2005: 11). They refer to the view that for an object to be present, it depends on a series of absences, elements which cannot be seen or known — ‘an object is a pattern of presences and absences’ (*loc cit*). They provide the example of the construction of an aircraft wing, whose form is composed partly of elements which are not physically present, such as the enemy air force, the body of the pilot, and the air pressure when flying at a particular altitude. For them:

Such objects are transformative, but the transformations are not the gentle flows discussed above in fluid objects. They are more like some of the differences mentioned by Mol. This is because they take the form of jumps and discontinuities. In this way of thinking then, constant objects are energetic, entities or processes which that juxtapose, distinguish, make and transform absences and presences. They are made in disjunction … we will talk of such entities as fire objects. (Law and Singleton 2005: 13)

This rests on the notion of fires as transformative, and dependent on difference, such as between fuel which is absent, and flame which is present. ‘Fire objects, then, depend upon otherness, and that otherness is generative.’ (*loc cit*) They apply this notion to an extended analysis of alcoholic liver disease, taking in multiple facets of the phenomenon, and its multiple ontology across different treatment sites. For them ‘[i]t is an object in the form of a dancing and dangerous pattern of discontinuous displacements between locations that are other to (but linked with) each other’ (*loc cit*). What distinguishes it from a *fluid object* is its inherent discontinuity which ‘…lives in and through juxtaposition of uncontrollable and generative otherness’ (*loc cit*).

## Postdigital Education as a *Fire Object*

The foregoing analysis is clearly about a very different context than postdigital education. However, I would propose that a *fire object* analysis might allow us further theoretical purchase on the ontology of teaching, engaging and communicating via screens and digital technologies, using video platforms such as Zoom or Teams. The operation of video calls or broadcasts inherently depends on difference and otherness, the absence of the interlocutor and the presence of oneself and the physical device, and associated sociomaterial assemblage. In simple terms, it would be nonsensical, or at least unusual, for this phenomenon to take place in a situation of embodied co-presence, as it is defined by absence. In addition, it is characterised by the lack of fluidity in these terms; the listener or speaker is either ‘on the call’ or not, although the form of presence may vary if video or audio only is used. This form of engagement is characteristic of alterity, a form of simultaneous absence and presence, in which one is both ‘there’ and ‘not there’. Like fire, it has a flickering ontology, with sudden flares of visual presence, loss of video or sound, and could perhaps also be likened to smouldering embers when participants are ‘there’, but with the mute button on and the video off. It is, like the disease described above, inherently discontinuous, partial, flickering, and multiple in its nature. The ‘star pattern’ also seems apposite as a means by which to conceptualise postdigital education, describing the experience of being present in a space, in a relationship of conjoined alterity with multiple others via a video platform such as Zoom.

## Fire Objects, Goffman’s *Selves* and *Forms of Talk*

Working with this reading, a connection might be made with early theorisations of the nature of the lecture, drawing on the work of Goffman, in particular his (1981) *Forms of Talk*. This is relevant in that allows for the previous *fire object* analysis to connect with a framing of the nature of teaching speech and *selves* which can also be understood in terms of absence, presence and alterity. In his essay ‘The Lecture’, Goffman sets out a view of the lecturing self as essentially multiple:

At the apparent center will be the textual self, that is, the sense of the person that seems to stand behind the textual statements made and which incidentally gives these statements authority. Typically, this is a self of relatively long standing, one the speaker was involved in long before the current occasion of talk. This is the self that others will cite as the author of various publications, recognise as the holder of various positions, and so forth… And he (sic) is seen as the ‘principal’ namely, someone who believes personally in what is being said and takes the position that is implied in the remarks. (Goffman 1981: 173)

This *textual self* comes into being in the preparation of the lecture materials, while the *animator self* emerges when the lecture is delivered, a being who Goffman refers to as a ‘talking machine’ (Goffman 1981: 171). In Goffman’s framing, there are three forms of talk; *memorisation*, *aloud reading*, and *fresh talk*. The latter he characterises as an illusion of spontaneity, as part of a performative display (see Friesen 2017 for an extensive discussion of Goffman and a historical review of technology in education).

The question for this paper is how these categories might intersect with a *fire object* analysis of postdigital education (for the purposes of this paper I am focusing on synchronous teaching online, see Gourlay (2021) for a Goffmanian analysis of the ‘flipped classroom’). I would propose that this reading could be extended to take in the lecturer selves and forms of talk in Goffman’s terms, as they could also be characterised in terms of absence, presence, and alterity. The textual self exists prior to the live event of the lecture or class. In this regard, it is characterised by the absence of the students, and of some elements of the technology such as Zoom, which is not generally used at this stage. Instead, the lecturer may be working with PowerPoint or other textual/multimodal semiotic online resources. Their voice and forms of talk are also absent at this stage. The textual artefact that is created also has the features of a *fire object*, as once complete, it is no longer fluid, but can either be absent or present in a flickering, fire-like manner, made present by sharing the screen during the live class online. The *animator self* is also fire-like; the lecturer emerges into being presence on the screens of the students in the form of a live video image or audio facility.

The lecturer’s subjectivity, it might be argued, is fundamentally made *other* in a condition of alterity, in a circumstance in which the students have never met that person face-to-face. The forms of talk may also be analysed in these terms; *memorisation* of blocks of text is defined by the absence of the text itself at the time of the animator self’s performance, while *aloud reading* is defined by the presence of the text and also the listeners, a form of gathering. *Fresh talk* is arguably an element of the lecture defined by alterity, in that it entails a form of layered performance which enrols the lecturer and students into subject positions which are essentially double; they are both entwined in a formal educational encounter governed by relevant generic conventions, while also participating in a more personal exchange or display, with the lecturer as an apparently spontaneous speaker, an element of side commentary which might be compared to marginalia in a Medieval text. In an online context, this might also include the type of procedural talk required in order to facilitate the class — such as the oft-repeated ‘Can you hear me?’, ‘Let me just share my slides now’, ‘Hopefully you can see that screen now’, and the ubiquitous ‘You’re on mute’. The status of the chat room is another area which might be included as a further category of ‘talk’, plus the presence on one’s own live image on screen while talking, although these elements are beyond the scope of this paper.

## Conclusions: It's Just Not the Same

In this paper, I have argued that the construct of ‘learning space’, when applied to the physical campus, is inadequate as a theorisation of the sociomaterial complexities of student engagement, and is reproductive of a very particular discourse of performative and observable interaction, underpinned by implicit notions of Biesta’s ‘learnification’, and also of the student as customer. I went on the explore concepts of space developed in science and technology studies, in particular the construct of *fire space*, arguing that this captures the flickering nature of absence, presence, and alterity in postdigital education, looking at the example of the synchronous lecture. I went on to suggest that this analysis may be rendered more granular with the inclusion of Goffman’s lecturing *selves* and associated *forms of talk*.

Clearly, Goffman’s framework was developed in a time prior to postdigital teaching. However, I would propose that it not only pertains to contemporary online teaching, it might also allow us to unpick the elements of talk that are required; the model could in fact be refined and extended further to reflect the changes brought about by digital mediation. I also contend, as set out above, that this set of *selves* and *forms of talk* are characteristic of *fire space* as discussed, and reinforce the analysis of postdigital teaching as a *fire object*. The framing of online teaching as taking place in *fire space* may have some implications in terms of how we see these events, touching on teaching practices and expectation of lecturers and students. One outcome might be a recognition of the flickering, unstable, and fundamentally uncanny nature of Zoom and other video platforms, allowing us to extricate ourselves from what might be termed discourses of replication, in which (arguably futile) attempts are made to recreate the conditions of the face-to-face embodied, ephemeral encounter. Arguably, this is doomed to failure, as can be hinted at the often-expressed view that ‘It’s just not the same’. It is clearly ‘not the same’ in terms of fostering a sense of connection, immediacy, and the ability ‘to read the room’, in a setting where the nuances of embodied performance, facial expression, gesture, and gaze cannot be fully deployed, in addition to the more subtle yet vital effects of physical copresence and ephemerality, the latter being compromised or at least complicated, as the digital lecture is recorded. Instead, the speaker must treat the screen as form of portal for digital performance (Gourlay 2020). In these respects and possibly others, this analysis allows us to theorise the many ways in which ‘it’s just not the same’, while the event may appear superficially similar in certain simulacrum-like respects. In terms of implications for teaching and student engagement, this reinforces the importance of enhancing of participants’ sense of relationality, connectedness, and inclusion in other ways and in other forums, particularly in a context where all engagement is online, such as in the current Covid-19 crisis (Gourlay et al. 2021). This is a matter of particular urgency in a context where moves towards greater use of online formats in higher education are being discussed as somehow ‘inevitable’, post-pandemic. I would argue, in conclusion, that it is all the more incumbent on the field therefore to recognise the fundamentally othering, even alienating nature of the online educational encounter in this uncanny, flickering, shifting *fire space*.
